# Single Cell RNA Sequencing Identifies Subsets of Hepatic Stellate Cells and Myofibroblasts in Liver Fibrosis

**DOI:** 10.3390/cells8050503

**Published:** 2019-05-24

**Authors:** Oliver Krenkel, Jana Hundertmark, Thomas P. Ritz, Ralf Weiskirchen, Frank Tacke

**Affiliations:** 1 Department of Medicine III, University Hospital Aachen, D-52074 Aachen, Germany; okrenkel@ukaachen.de (O.K.); jhundertmark@ukaachen.de (J.H.); tritz@ukaachen.de (T.P.R.); 2 Boehringer-Ingelheim Pharma GmbH & Co. KG, D-88397 Biberach an der Riss, Germany; 3 Department of Hepatology/Gastroenterology, Charité University Medical Center, D-13353 Berlin, Germany; 4 Institute of Molecular Pathobiochemistry, Experimental Gene Therapy and Clinical Chemistry (IFMPEGKC), University Hospital Aachen, D-52074 Aachen, Germany; rweiskirchen@ukaachen.de

**Keywords:** hepatic stellate cells, myofibroblasts, liver fibrosis, scRNASeq

## Abstract

Activation of hepatic stellate cells (HSCs) and their trans-differentiation towards collagen-secreting myofibroblasts (MFB) promote liver fibrosis progression. During chronic liver disease, resting HSCs become activated by inflammatory and injury signals. However, HSCs/MFB not only produce collagen, but also secrete cytokines, participate in metabolism, and have biomechanical properties. We herein aimed to characterize the heterogeneity of these liver mesenchymal cells by single cell RNA sequencing. In vivo resting HSCs or activated MFB were isolated from C57BL6/J mice challenged by carbon tetrachloride (CCl_4_) intraperitoneally for 3 weeks to induce liver fibrosis and compared to in vitro cultivated MFB. While resting HSCs formed a homogenous population characterized by high platelet derived growth factor receptor β (PDGFRβ) expression, in vivo and in vitro activated MFB split into heterogeneous populations, characterized by α-smooth muscle actin (α-SMA), collagens, or immunological markers. S100 calcium binding protein A6 (S100A6) was a universal marker of activated MFB on both the gene and protein expression level. Compared to the heterogeneity of in vivo MFB, MFB in vitro sequentially and only transiently expressed marker genes, such as chemokines, during culture activation. Taken together, our data demonstrate the heterogeneity of HSCs and MFB, indicating the existence of functionally relevant subsets in hepatic fibrosis.

## 1. Introduction

Hepatic stellate cell (HSC) activation and their trans-differentiation to myofibroblasts (MFB) due to chronic hepatic inflammation is a major hallmark feature of liver fibrosis [1]. Preventing or reversing excessive hepatic scarring is a major therapeutic target in treating chronic liver diseases, such as viral hepatitis and alcoholic and non-alcoholic steatohepatitis [2]. Resting HSC store lipids, such as retinol, can become activated following triggering signals released by damaged hepatocytes or activated local immune cells, such as e.g., Kupffer cells. Activating signals include transforming growth factor-β (TGF-β), platelet derived growth factors as well as various cytokines, such as interleukin-1β (IL-1β), and tumor necrosis factor-α (TNF-α) [3]. Activated MFB alter the composition and density of the extracellular matrix, by secreting collagens, and release inflammatory mediators, including chemokines and cytokines, thereby aggravating local inflammation. Interestingly, a variety of different functions have been assigned to HSCs and/or MFB, ranging from extracellular matrix production, mechanical properties (e.g., contraction and vascular resistance regulation in the liver), and lipid metabolism to immune regulation [4], raising the question about yet unrecognized, functionally diverse subsets of HSCs/MFB. The aim of this study was therefore the evaluation of the heterogeneity of resting HSCs and activated MFB, both in vivo and in vitro, by single cell RNA sequencing (scRNASeq) analysis. We found that resting platelet derived growth factor receptor β- (PDGFR-β) positive HSCs show a high homogeneity, while activated α-smooth muscle actin- (α-SMA) positive MFB split into four different subpopulations, characterized by uniquely expressed gene patterns related to collagen synthesis or immunologic functions. We could identify S100 calcium binding protein A6 (S100A6) as a key marker of activated MFB. Furthermore, we found that in vitro activated MFB lack key functions of in vivo activated MFB as the production of various chemokines, such as CC-chemokine ligand 2 (CCL2) and CXC-chemokine ligand 1 (*CCL1*).

Taken together, our data demonstrate the heterogeneity of activated MFB in vivo and highlight the differences of in vivo and in vitro activated MFB, leading to a better understanding of HSCs to MFB trans0differentation during liver fibrosis.

## 2. Materials and Methods

### 2.1. Animal Models and Induction of Liver Fibrosis

C57Bl6/J mice were housed under specific pathogen free conditions in the animal facility of the University Hospital, Aachen. All experiments that are described were approved by the corresponding legal authorities (Landesamt für Natur, Umwelt und Verbraucherschutz NRW, LANUV NRW). To induce hepatic fibrosis, mice received intraperitoneal injections of 0.5 mL per kg bodyweight carbon tetrachloride (CCl_4_) three times per week for a total of three weeks. Controls received an equivalent amount of oil. After three weeks, mice were euthanized and analyzed as detailed below.

### 2.2. Isolation of Ultrapure Hepatic Stellate Cells by Flow Cytometric Sorting

Ultrapure HSCs were isolated from the liver of healthy C57Bl6/J mice by collagenase digestion and differential gradient centrifugation, followed by fluorescence activated cell sorting (FACS) purification for UV autofluorescence as described before [5]. In detail, the liver was perfused via the *Vena portae* with a prewarmed perfusion HEPES buffer to remove remaining blood from the tissue. the liver was then perfused with 0.5 mg/mL pronase E (Merck, Darmstadt, Germany) and 0.75 U/mL collagenase P (Roche, Basel, Switzerland) for 4.5 min each. The liver was then removed and additionally digested at 37 °C in a water bath for another 20 min. After filtering via a 40 µm cell strainer, HSCs were purified by ultraviolet autofluorescence by using a BD FACS Aria II SORP Cell Sorter (BD Biosciences, Franklin Lakes, NJ, USA).

### 2.3. Cultivation of Hepatic Stellate Cells

4 × 10^5^ purified HSCs were seeded on an uncoated 6 well plate in Dulbecco’s Modified Eagle Medium (DMEM) with 10% heat inactivated fetal calf serum (FCS) and 1% penicillin/streptomycin. After one, three, seven, or nine days, cells were then detached by accutase treatment for 10 min. Afterwards, the detached cells were washed once with cold phosphate-buffered saline (PBS) and pelleted by centrifugation at 570 rcf for 5 min in a cold centrifuge. Cells were then resuspended at 500 cells per µl in cold PBS with 0.1% bovine serum albumin (BSA) and directly subjected to the single cell RNA sequencing analysis, according to the manufacturers protocol.

### 2.4. Isolation of Liver Non-Parenchymal Cells

Livers were perfused with cold PBS, followed by digestion for 40 min at 37 °C with 100 µg/mL Collagenase D and 50 µg/mL DNase I (Worthington Biochemicals, Lakewood, NJ, USA). Digestion was stopped by adding cold HBSS with 0.1 mM EDTA. Single cell suspension was obtained by using a 40 µm cell strainer. After washing once with cold PBS, liver non-parenchymal cells were purified by 18% Nycodenz gradient centrifugation. Obtained cells were then stained with CD31-FITC and CD45-APC-Cy7 (BD Biosciences, Heidelberg, Germany). Retinol droplets were measured as autofluorescence by UV-laser excitation. Dead cells were excluded by Hoechst 33342 staining (Sigma-Aldrich, Taufkirchen, Germany).

### 2.5. Single-Cell RNA Sequencing

Freshly isolated cells, or in vitro cultivated MFB, were analyzed by using the Chromium Single Cell 5′ kit (10× Genomics, Pleasanton, CA, USA), according to manufacturer’s protocol. In detail, cells were resuspended at 500 cells per µL in sterile filtered cold PBS containing 0.1% BSA. The experiment was conducted for 5000 recovered cells. After, library generation sequencing was performed by Illumina sequencing on a NextSeq 550 (IZKF genomics facility of the RWTH Aachen University, Aachen, Germany) as detailed before [6]. Primary analysis was done by using an in-house pipeline based on “cellranger” (10× Genomics). Additional analysis was then performed by using the “Seurat” (v2.3.2) [7] package for R (v3.5) (https://www.r-project.org/). Cluster identification was based on the 50 most significant principal components.

### 2.6. Immunohistochemistry

Immunohistochemistry was performed on formalin-fixed and paraffin-embedded (FFPE) liver sections for α-smooth muscle actin (α-SMA) (clone ASM-1/1A4; Sigma-Aldrich, Taufkirchen, Germany), platelet derived growth factor-β (PDGFR-β) (clone 42G12; Abcam, Cambridge, UK), and S100 calcium binding protein A6 (S100A6) (clone EPNCIR121; Abcam). All primary antibodies were diluted 1:100. For immunofluorescence, secondary goat anti-mouse Cy5 (Abcam) and goat anti-rabbit Al488 (Abcam) were used at a dilution of 1:200. Nuclei were stained with DAPI (Sigma-Aldrich, Taufkirchen, Germany). Micrographs were taken using an Axio Observer Z1 equipped with an Axio Cam MR (Zeiss, Oberkochen, Germany)

## 3. Results

### 3.1. Single Cell RNA Sequencing Identifies Four Different Clusters of Myofibroblasts

**Figure 1:**
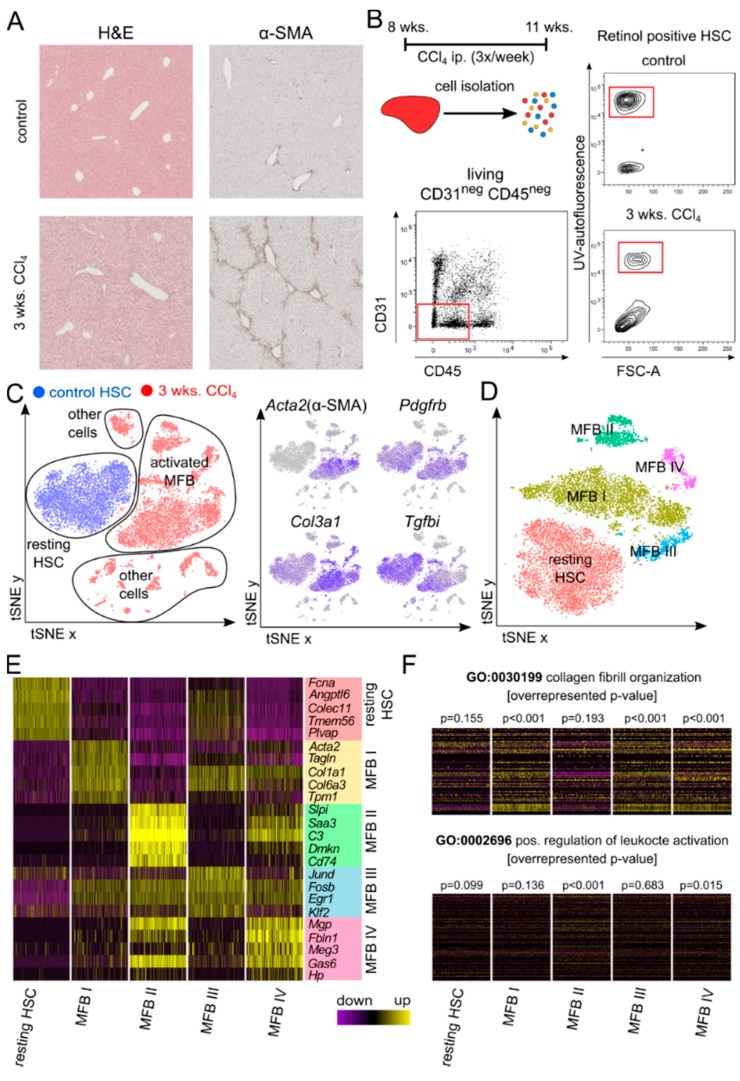
Identification of four sub-populations of activated myofibroblasts (MFB) by single cell RNA sequencing (scRNASeq). (**A**) Representative images of formalin fixed and paraffin-embedded hematoxylin and eosin (H&E) or anti-α-smooth muscle (α-SMA) stained liver sections of control mice and mice subjected to repetitive carbon tetrachloride (CCl_4_) injections for 3 weeks. (**B**) Treatment scheme of mice during CCl_4_ treatment. Gating strategy for the isolation of resting hepatic stellate cells (HSCs) from healthy mice and activated MFB from CCl_4_ treated mice by FACS. (**C**) t-distributed stochastic neighbor embedding (t-SNE) plots mapping the identity of cells to resting HSCs (blue) and MFB from CCl_4_ treated liver (red), and the expression of marker genes used for identifying HSCs and MFB. (**D**) Definitive subset clustering of resting HSCs and activated MFB after exclusion of contaminating cells from scRNASeq data sets. (**E**) Log fold change (avg-logFC) gene expression of the top five marker genes for each cluster. For a better comparability, the number of cells in each cluster is aligned. (**F**) Avg-logFC gene expression of genes in the corresponding Gene Ontology (GO) categories, with overrepresented p-value in each cluster. *n* = 4 with an average of 5000 cells per condition and ~60,000 reads per cell.

Chronic liver injury involves the activation of HSCs and their subsequent transformation towards collagen secreting MFB. To assess the heterogeneity of activated MFB, we isolated liver non-leukocytes non-parenchymal cells from three weeks-CCl_4_-treated mice and rested HSCs from untreated control mice. The presence of liver fibrosis after three weeks of CCl_4_ treatment was confirmed by a hematoxylin and eosin (H&E) stain as well as α smooth muscle actin (α-SMA) immunohistochemistry on FFPE tissue sections (Figure 1A). To capture all potential hepatic MFB from fibrotic livers, we excluded CD31 positive endothelial cells as well as CD45 positive leukocytes, but subjected all remaining double negative cells to scRNASeq analysis. FACS purified retinol positive HSCs, isolated from livers of untreated mice, served as a control (Figure 1B). Both HSCs and MFB were identified by their expression of platelet derived growth factor receptor-β (*Pdgfrb*), while the expression of alpha smooth muscle actin (*Acta2*), collagen, type III, alpha 1 (*Col3a1*)*,* and transforming growth factor, beta induced (*Tgfbi*) allowed the distinction of differently activated states of HSCs and MFB (Figure 1C).

In the next step, we excluded contaminating endothelial cells, leukocytes, and hepatocytes from our dataset to identify clusters of resting HSCs and activated MFB. We found that the HSCs form a highly homogenous cluster, while the MFB separated into four different sub-clusters, which we termed MFB I to IV (Figure 1D). Analysis of the most significantly expressed marker genes for each cluster allowed the further functional differentiation of these subtypes (Figure 1E). Besides common markers, such as PDGFR-β or various collagens, resting HSCs were uniquely characterized by a high expression of ficolin A (*Fcna*), which has been described to trim extracellular collagen as well as being an activator of a lectin complement pathway, and the hepatokine (*Angptl6*), an inducer of energy expenditure and regulator of the expression of fibroblast derived growth factor 21 (*Fgf21*) in white adipose tissue [8,9].

The major population of activated MFB, termed MFB I, was defined by a high expression of *Acta2*, the smooth muscle cell specific cytoskeletal protein, transgelin (*Tglna*), as well as various types of collagens, such as *Col1a1*, *Col3a1*, or *Col6a3*. The second cluster, MFB II, expressed less extracellular matrix associated genes, but did express the inflammation associated serum leukocyte protease inhibitor (*Slpi*), complement factor *C3* (*C3*), serum amyloid A3 (*Saa3*), and cluster of differentiation 74 (*Cd74*). These data indicate towards the existence of a distinct subset of immunoregulatory MFB, characterized by a reduced capacity of modulating the extracellular matrix. The subset, MFB III, comprised proliferating fibroblasts, indicated by an expression of components of the activator protein 1 (*Ap1*) transcription factor, such as anti-apoptotic jun D (*Jund*) and its dimer forming partner FBJ osteosarcoma oncogene B (*FosB*). The smallest subset, MFB IV, displayed a mixed phenotype with a high expression of extracellular matrix modulators, such as the matrix gla protein (*Mgp*) and fibulin 1 (*Fbln1*), as well as growth arrest specific 6 (*Gas6*). Some of these marker genes have been described for portal fibroblasts [10]. While clusters MFB I, III, and IV showed a high expression of genes associated with the Gene Ontology (GO) category collagen fibril organization and extracellular matrix buildup (Figure 1F), cluster MFB II expressed less matrix associated genes, and more genes associated with the GO category positive regulation of leukocytes and immune regulation (Figure 1F). Due to the combination of characteristics of myeloid leukocytes, as well as of myofibroblasts, cluster MFB II most likely includes trans-differentiated myeloid myofibroblasts, which have been described before [11].

### 3.2. S100A6 Represents a Marker for Activated Myofibroblasts

**Figure 2:**
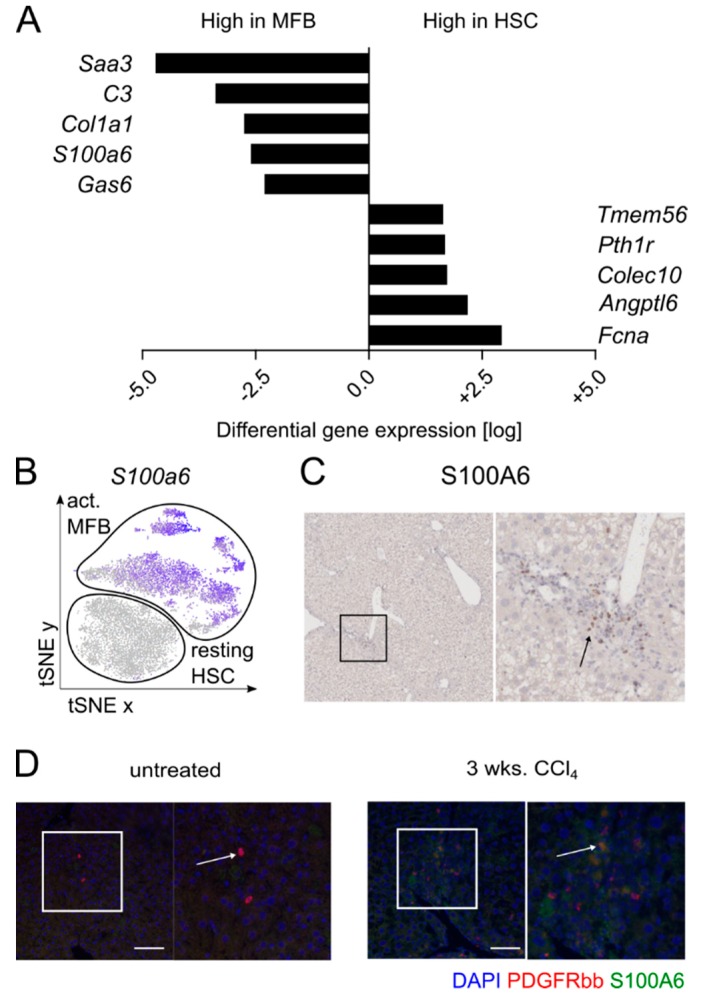
The S100 calcium binding protein A 6 (S100A6) expression marks activated myofibroblasts. (**A**) Differential gene expression analysis of HSCs versus MFB, showing the top five genes upregulated in both groups. (**B**) t-SNE plot of relative gene expression of S100A6, based on scRNASeq analyses from normal and fibrotic mouse livers. (**C**) Representative image of immunohistochemistry staining for S100A6 in CCl_4_-treated fibrotic liver. (**D**) Representative images of immunofluorescence co-staining for PDGFR-β (red) and S100A6 (green) on untreated and CCl_4_-treated fibrotic liver. Nuclei are stained with DAPI (4′,6-diamidino-2-phenylindole, blue). Scale bar represents 100 µm.

At present, α-SMA is largely accepted as a marker of activated MFB [12]. However, in our scRNASeq analysis, this marker only recognized a subset of activated MFB (see Figure 1C). We therefore wanted to identify markers that are uniquely and uniformly upregulated on all subsets of activated MFB in vivo (Figure 2A). We found that the S100 calcium binding protein A 6 (*S100a6*) is highly upregulated on activated MFB but not on resting HSCs (Figure 2A,B). We first confirmed the presence of S100A6 positive cells in the periportal (i.e., fibrotic) areas of CCl_4_-treated livers by immunohistochemistry (Figure 2C). By using immunofluorescence co-staining for PDGFR-β (red) and S100A6 (green) we could then confirm the presence of double positive cells, corroborating the MFB phenotypes observed by our scRNASeq analysis (Figure 2D).

### 3.3. Differential Expression of Chemokines and Collagens by Activated Myofibroblast Subsets

**Figure 3:**
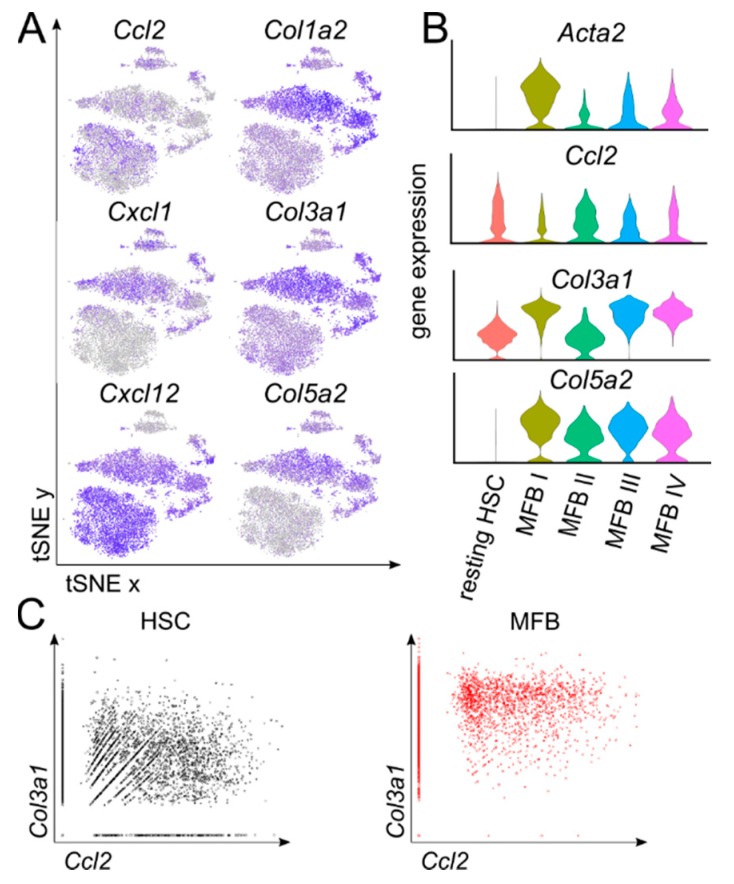
Differential chemokine and collagen gene expression patterns in hepatic stellate cells and myofibroblasts. (**A**) Feature plots showing the relative gene expression strength of selected marker genes. (**B**) Violin plots showing the relative gene expression of activation markers. (**C**) Gene plots for the normalized gene expression of *Col3a1* and *Ccl2*. *n* = 4 with an average of 5000 cells per condition and ~60,000 reads per cell.

Upregulated expression of chemokines, such as CCL2, CXCL1, or CXCL12, and collagens, particularly COL1A2, COL3A1, or COL5A2, are often named attributes for activated MFB [13]. We herein wanted to analyze whether different subsets of either chemokine or collagen-producing MFB sub-clusters exist in fibrotic livers in vivo (Figure 3A). By scRNASeq analysis, the expression of collagens is homogenously upregulated in activated MFB, while chemokines show a more restricted pattern. While the monocyte recruiting chemokine *Ccl2* is expressed by both resting HSCs and MFB, neutrophil recruiting *Cxcl1* is strongly associated with activated MFB only. *Cxcl12,* on the other hand, is highly expressed by both HSCs and MFB, except for cluster MFB II, representing myeloid myofibroblasts (Figure 3A,B and Supplementary Table S1). By correlating the expression of the chemokine *Ccl2* with *Col3a1* on a single-cell level, resting HSCs comprised cells that express only *Col3a1*, cells co-expressing *Col3a1*, or cells that only express *Ccl2*. Interestingly, activated MFB could be differentiated by their expression of *Ccl2*, while all cells highly expressed *Col3a1* (Figure 3C). These data confirm the universal importance of an upregulated collagen expression during the activation of HSCs to MFB, while resting HSCs and selected MFB clusters demonstrate the capacity to secrete chemokines and thereby modulate the inflammatory environment in their surroundings.

### 3.4. In Vitro Activated MFB Share Key Characteristics of In Vivo MFB

**Figure 4:**
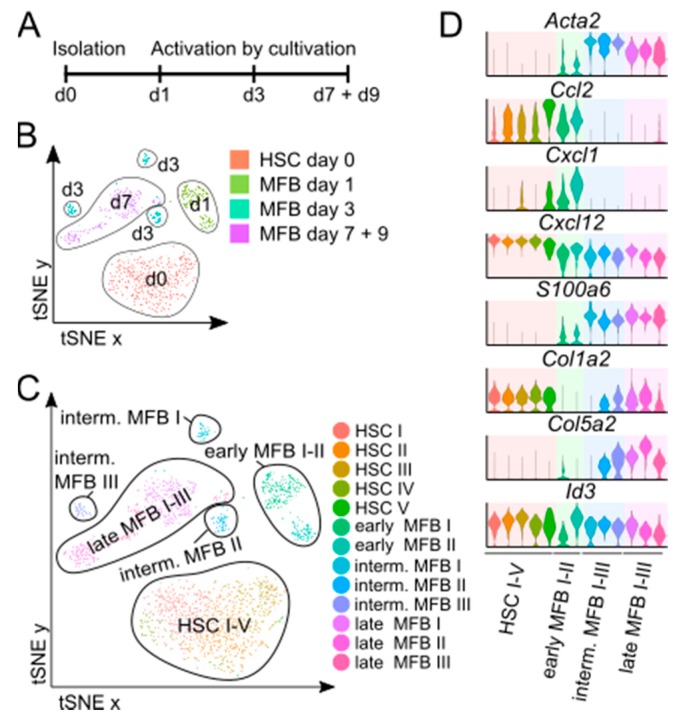
Single-cell RNA sequencing analysis of in vitro activated myofibroblasts. (**A**) Schematic overview of the experimental setup. (**B**) t-SNE plot showing all in vitro activated MFB and resting HSCs dependent on their origin. (**C**) t-SNE plot showing all in vitro activated MFB and resting HSCs dependent on cluster. (**D**) Violin plots showing the relative expression of selected marker genes for each cluster. *n* = 4 with an average of 1000 cells per condition.

Our scRNASeq analyses revealed a striking heterogeneity of MFB in vivo compared to resting HSCs, indicating a functional diversity of these cells during fibrogenesis in vivo. This prompted us to investigate to which extent in vitro activated MFB would reflect this heterogeneity as well. Ultrapure FACS-sorted mouse HSCs were therefore cultivated for up to 9 days on uncoated plastic dishes, the standard model of HSCs to MFB trans-differentiation in vitro [14]. HSCs and MFB were harvested at baseline, day 1, day 3, and days 7 and 9 for scRNASeq analysis (Figure 4A). On the one hand, scRNASeq analysis revealed that in vitro activated MFB clustered dependent on the time of cultivation and could be differentiated into early (day 1), intermediate (day 3), and late (day 7 and day 9) MFB (Figure 4B,C). On the other hand, in vitro activation induced a remarkable MFB heterogeneity over time, which became apparent at the intermediate (day 3) and the late (days 7 and 9) time-point. A more granular analysis of the scRNASeq data generated from the culture-activated HSCs/MFB revealed sub-clusters at all time-points (Figure 4C), for which the marker gene expression patterns varied (Figure 4D and Supplementary Table S2).

We then particularly looked at some markers that we had identified from the in vivo data sets (compare to Figure 2 and Figure 3). The expression of α-SMA (*Acta2*) and *S100a6* was found to be upregulated from early to late MFB in vitro, indicating the relevance of *S100a6* expression as a marker of activated MFB and revealing a high concordance with the in vivo data. For collagens, we observed a clear upregulation of *Col1a2* or *Col5a2* over time during MFB maturation in vitro (Figure 4D). On the contrary, chemokine expression tended to decrease in sequential samples during culture-induced MFB activation. The production of the chemokines *Ccl2* and *Cxcl1* was only found in early MFB, while late MFB populations from day 3 to day 9 did not show any expression of these chemokines. *Cxcl12* displayed the highest expression in resting HSCs and early MFB (Figure 4D). While in vivo, MFB from fibrotic livers consisting of heterogeneous subsets, in which either collagens or chemokines were highly expressed, in vitro activated MFB only expressed these marker genes in a time-dependent manner and not simultaneously, indicating in vitro activation reflected important aspects of MFB biology only transiently during cultivation.

## 4. Discussion

HSCs trans-differentiation to MFB is a key event in the progression from chronic liver injury to liver fibrosis, which occurs as a consequence of chronic hepatic inflammation e.g., following alcoholic or non-alcoholic steatohepatitis. Liver fibrosis is also considered as a cornerstone event in the progression toward liver cirrhosis or hepatocellular carcinoma [15]. Given the wide range of functional contributions of HSCs and MFB for liver physiology and for fibrogenesis [4], a better understanding of the heterogeneity and subpopulations of HSC and MFB may help to identify novel therapeutic targets to treat liver fibrosis. The upregulation of collagens and α-SMA in MFB is a well-established finding [16] and was confirmed by our single-cell based data for MFB in vivo as well as in vitro. While almost all was MFB upregulated collagen production, only half of the cells expressed chemokines. This was even more striking in vitro, in which chemokine expression appeared early after HSC activation and was down-regulated during later maturation. Activated MFB secrete chemokines capable of recruiting myeloid cells from the circulation, such as e.g., neutrophils via CXCL1 and monocytes via CCL2, which has been linked to exacerbated hepatic inflammation [3]. In fact, HSCs had been reported to induce monocyte chemotaxis via CCL2 upon recognizing danger signals via toll-like receptor 4 [17]. Our scRNASeq data support that this is a feature of HSCs and early activated MFB in vitro, while the existence of MFB sub-populations in fibrotic livers in vivo allows to maintain the production of inflammatory chemokines and cytokines during fibrogenesis. On the other hand, as this feature was missing in our in vitro MFB dataset for the late MFB, we conclude that the use of plastic adherence cultivated MFB may not be a useful tool for analyzing chemokine production by MFB when the cells are being studied after 7 days of culture. However, it needs to be noted that we did not further evaluate other stimuli or cultivation methods as TGF-β or platelet derived growth factor subunit beta (PDGF-β) for the in vitro culture, which could potentially give different results regarding collagen and chemokine expression.

The scRNASeq data sets generated in this work may help to guide future studies on the functional relevance and interactions of HSC/MFB subsets. For instance, we identified S100A6 as a novel marker of activated MFB, following liver fibrosis, which could be confirmed in vitro. The functional involvement of S100A6 for HSCs/MFB signaling, however, requires further evaluation. Data from mouse models of fibrosis indicated that recombinant S100A6 would aggravate fibrogenesis via inducing HSCs proliferation [18]. While S100A6 was consistently found across HSCs/MFB in vivo and in vitro, some MFB properties appear restricted to distinct clusters and/or differentiation states. Cluster MFB II showed characteristics of both leukocytes by expressing CD74, *C3* or SLPI but also expressed various collagens. These cells might represent macrophages that have trans-differentiated into myofibroblasts, which has been described for renal fibrosis previously [11] and could potentially explain parts of the immunologic properties that have often been assigned to HSCs and/or trans-differentiated MFB [4].

Importantly, more work is needed to get a better spatial resolution on the MFB populations in fibrosis. The cluster MFB IV showed some features that had been previously reported for portal fibroblasts [19]. It will be important to confirm the scRNASeq data in other models of liver fibrosis, such as bile duct ligation leading to a preferential expansion of the portal fibroblasts, and to define the exact localization of the MFB sub-clusters in fibrotic livers in vivo. Last, but not least, it will be important to identify HSC and MFB populations in human liver. scRNASeq data from healthy human liver already indicated the existence of HSC clusters [20], and similar analyses from cirrhotic human livers are currently ongoing.

Taken together, our scRNASeq analyses from healthy and fibrotic mouse livers demonstrated a yet unrecognized heterogeneity of HSCs and MFB in vivo, suggesting a concerted interplay of functionally diverse MFB subsets during liver fibrogenesis.

## Supplementary Materials

The following are available online at https://www.mdpi.com/2073-4409/8/5/503/s1, Table S1: Mean gene expression of HSC and MFB I-IV, Table S2: Mean gene expression of HSC I-V and early, intermediate and late MFB.
