# Supplementary material for: Single Cell RNA Sequencing Identifies Subsets of Hepatic Stellate Cells and Myofibroblasts in Liver Fibrosis
Source: Cells. 2019 May 24;8(5):503. doi: 10.3390/cells8050503 (PMC6562512; doi:10.3390/cells8050503)
Supplement: Supplementary file 1 [file cells-08-00503-s001.pdf]

Supplementary Table S1. Mean gene expression of HSCs and MFB I–IV.

Gene Name	Resting HSCs	MFB I	MFB II	MFB III	MFB IV
ACTA2	0.255233	21.04447	1.443986	4.665647	4.39712
CCL2	13.99074	4.476969	8.330583	4.541892	7.580274
COL3A1	9.172187	67.12583	8.923302	64.67622	65.38038
COL5A2	0.564254	6.26082	2.894847	4.876634	3.744942

Supplementary Table S2. Mean gene expression of HSCs I–V and early, intermediate, and late MFB.

Gene Name	HSC I	HSC II	HSC III	HSC IV	HSC V	Early MFB I
ACTA2	0.14393771	0.36639035	0.01772177	0.11650378	0.45571147	3.75705383
CCL2	6.92935975	22.1482896	16.9932256	15.5947369	160.629936	24.5157773
CXCL1	0.19436314	0.54072211	2.9897711	0.46346092	5.96367083	5.9975138
CXCL12	73.223511	59.5265139	62.2957151	73.4734595	36.9866964	13.4771641
COL1A2	4.42562979	3.81501036	4.3317417	5.10468878	4.12737645	0.20235445
COL5A2	0.61716154	0.52265032	0.49232782	0.81247689	0.45051412	0.41222067
ID3	14.4257135	19.585539	27.7387347	11.2151014	18.9112755	2.17908022
S100A6	0.09875259	0.02769721	0.01167219	0.02387577	0	1.92864876

Gene Name	Early MFB II	Interm. MFB I	Interm. MFB II	Interm. MFB III	Late MFB I	Late MFB II	Late MFB III
ACTA2	1.65381662	55.3086314	55.7388147	56.6587938	19.5884047	22.6909218	20.0948946
CCL2	37.1024492	0.23365574	0.63213034	0.12451785	0.32242775	0.30031165	2.00785768
CXCL1	45.8722936	0.02300151	0.06263036	0.0064203	0.02521638	0.02640656	0.15475411
CXCL12	24.4427018	11.5119612	17.3555205	10.5522942	20.521856	10.0320541	8.98169019
COL1A2	0.50383921	0.2046156	1.16649636	2.64824904	3.00512838	4.55456308	1.37125665
COL5A2	0.31213778	0.91239069	2.37837071	5.42714586	6.91048757	20.0563126	3.80688843
ID3	28.9727643	8.78347231	12.2669527	8.21522514	11.2122989	4.83146325	4.12031423
S100A6	1.48484728	37.4252259	16.2693933	18.2021413	33.9949525	25.2679785	30.4729068
